# Genetic and Structural Variations in Czech Patients With Congenital Myopathies

**DOI:** 10.1111/cge.14782

**Published:** 2025-06-17

**Authors:** Jana Zídková, Barbora Lauerová, Lívie Mensová, Tereza Kramářová, Johana Kopčilová, Kamila Réblová, Magdaléna Soukup Vodičková, Martina Hujňáková, Jana Haberlová, Marie Rohlenová, Radim Mazanec, Jana Šoukalová, Renata Gaillyová, Emílie Vyhnálková, Miroslava Balaščaková, Pavlína Danhofer, Lenka Juříková, Dagmar Grečmalová, Andrea Gřegořová, Pavlína Plevová, Martina Langová, Tomáš Honzík, Martin Magner, Martina Klincová, Pavla Solařová, Mária Šenkeříková, Lenka Fajkusová

**Affiliations:** ^1^ Centre of Molecular Biology and Genetics University Hospital Brno and Masaryk University Brno Czech Republic; ^2^ Department of Paediatric Neurology 2nd Faculty of Medicine, Charles University and Motol University Hospital Praha Czech Republic; ^3^ Department of Neurology 2nd Faculty of Medicine, Charles University and Motol University Hospital Praha Czech Republic; ^4^ Laboratory of Functional Genomics and Proteomics National Centre for Biomolecular Research, Faculty of Science, Masaryk University Brno Czech Republic; ^5^ Central European Institute of Technology Masaryk University Brno Czech Republic; ^6^ Institute of Medical Genetics and Genomics University Hospital Brno and Masaryk University Brno Czech Republic; ^7^ Department of Biology and Medical Genetics 2nd Faculty of Medicine, Charles University and Motol University Hospital Prague Czech Republic; ^8^ Department of Child Neurology University Hospital Brno and Masaryk University Brno Czech Republic; ^9^ Institute of Clinical and Molecular Pathology and Medical Genetics University Hospital Ostrava Ostrava Czech Republic; ^10^ Institute of Laboratory Medicine Medical Faculty Ostrava Ostrava Czech Republic; ^11^ Department of Medical Genetics Thomayer University Hospital Praha Czech Republic; ^12^ Department of Paediatrics and Inherited Metabolic Disorders First Faculty of Medicine, Charles University and General University Hospital in Prague Praha Czech Republic; ^13^ Department of Pediatric Anesthesiology and Intensive Care Medicine University Hospital Brno and Masaryk University Brno Czech Republic; ^14^ Department of Medical Genetics University Hospital Hradec Králové Hradec Králové Czech Republic

**Keywords:** breakpoint analysis, congenital myopathy, deletion, structural variants

## Abstract

Congenital myopathies (CMs) are a heterogeneous group of genetic muscle disorders characterized by hypotonia and muscle weakness, with pathogenic variants identified in at least 41 genes and inheritance patterns including autosomal dominant (AD), recessive (AR), and X‐linked (XL). We present 79 unrelated patients with genetically confirmed CM using next‐generation sequencing (NGS). A total of 113 mutant alleles carrying 97 different variants with a presumed pathogenic effect were identified. According to the HGMD database, 54 of these variants have been reported exclusively in the Czech CM population to date. All but five variants were small‐scale. Large gene deletions were identified in the *MTM1*, *NEB*, and *RYR1* genes. Sequencing of breakpoint junctions in the identified *NEB* and *RYR1* deletions provided insights into the upstream mechanisms leading to genomic instability and resulting in structural variations. We present the family with dominant inheritance of the *NEB* deletion of exons 19–78. We assume that our family represents another reported case of a dominant mutation in the *NEB* gene. Our results contribute to further knowledge in the field of neuromuscular diseases and mutational mechanisms.

## Graphical abstract

We present 79 unrelated patients with genetically confirmed congenital myopathy (CM). A total of 113 mutant alleles carrying 97 different variants with a presumed pathogenic effect were identified. All but five variants were small scale. The mode of inheritance was autosomal dominant (AD) (44.3%), autosomal recessive (AR) (43.0%), and X‐linked (XL) (12.7%).

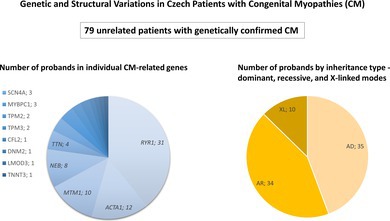

## 1 Introduction

Neuromuscular disorders (NMDs) comprise 1216 genetically determined diseases associated with 686 genes (www.musclegenetable.fr; updated January 17, 2024). This study focuses on congenital myopathies (CMs), a heterogeneous group of muscle disorders primarily characterized by hypotonia and muscle weakness, typically present at birth or emerging during infancy with a slow progression. The clinical spectrum ranges from severe neonatal forms with congenital arthrogryposis to milder childhood‐onset forms with nonprogressive muscle weakness. In neonatal and early‐onset cases, symptoms may include reduced fetal movements, arthrogryposis, severe hypotonia, feeding difficulties, and respiratory insufficiency. Normal respiratory function at birth does not preclude later‐onset respiratory complications. Additional early symptoms include congenital hip dislocation and hypomimia. As affected children grow, hypotonia remains stable, delaying motor milestones, and leading to musculoskeletal abnormalities such as joint contractures and scoliosis. Progressive muscle atrophy, low body weight, and impaired extrinsic eye movements (e.g., ptosis, strabismus, ophthalmoparesis) may also develop [1], [2].

Pathogenic variants in at least 41 genes have been implicated in CMs. These genes demonstrate various inheritance patterns, including autosomal recessive (AR), autosomal dominant (AD), and X‐linked (XL) modes. Notably, mutations in the same gene may result in both dominant and recessive inheritance [1]. Historically, CM classification has been based on histopathological features. Major subtypes include nemaline myopathy, core myopathy, centronuclear myopathy, myosin storage myopathy, and congenital fiber‐type disproportion (CFTD) [1]. The most frequently implicated genes include *NEB* and *ACTA1* in nemaline myopathies [3] and *RYR1* in core myopathies [4]. Patients diagnosed on the base of *RYR1* variants may be at risk for the development of malignant hyperthermia (MH). However, not all *RYR1* pathogenic variants infer MH risk [5]. XL centronuclear myopathies are caused by pathogenic variants in *MTM1*. Females carrying a heterozygous mutation are often asymptomatic but may also show mild weakness of late onset. AD centronuclear myopathies are primarily associated with *DNM2* [6]. *MYH7* pathogenic variants underlie myosin storage myopathy. CFTD myopathy is most commonly caused by pathogenic variants in *TPM3* and *TPM2* [7], [1].

Advancements in next‐generation sequencing (NGS), including targeted gene panels, whole‐exome sequencing, and whole‐genome sequencing (WGS), have revolutionized CM diagnosis by facilitating the rapid identification of pathogenic variants. Given the often nonspecific histopathological findings, genetic testing has increasingly replaced muscle biopsy as the primary diagnostic approach. Consequently, CM classification is shifting toward a genotype‐based framework.

This study provides a comprehensive molecular characterization of a large cohort of Czech patients with CM, encompassing cases with disease onset from the prenatal period to preschool age. The research was conducted in collaboration with neuromuscular centers and medical genetics departments across the Czech Republic.

## 2 Materials and Methods

### 2.1 Targeted NGS

To identify sequence variants associated with neuromuscular disorders (NMDs), we utilized the KAPA HyperChoice capture method (Roche) followed by NGS on the NextSeq 500 platform (Illumina). Custom capture probes were designed to target exons and adjacent intronic regions of 686 genes implicated in NMDs (www.musclegenetable.fr). Sequencing reads were aligned to the human genome reference hg19/GRCh37, and variant calling was performed using CLC Genomics Workbench (Qiagen). The analysis aimed to detect both small‐scale sequence variants and large gene deletions/duplications by assessing copy number variations (CNVs). In the CNV analysis, read mapping of the healthy control from the sequencing run was set as reference, and read mappings of patient samples were compared against it in a statistical framework. Amplification and deletion annotations in the targeted regions were produced.

Identified variants were classified based on the ACMG guidelines [8] and further evaluated using the VarSome variant classifier (https://varsome.com). Selected variants were validated using PCR and Sanger sequencing with the BigDye Terminator Cycle Sequencing Kit (Applied Biosystems) on the ABI 3130xl Genetic Analyzer (Applied Biosystems). Segregation analysis was performed by sequencing family members to assess inheritance patterns.

This study included all unrelated patients genetically diagnosed with CM at the Centre of Molecular Biology and Genetics (CMBG), University Hospital Brno, since 2012. As the sole molecular diagnostic center for CMs in the Czech Republic, the CMBG dataset represents the Czech CM population. Only unrelated patients with a clinical suspicion of CM and presumed pathogenic variants (biallelic in AR genes or monoallelic in AD genes) were included in the study. The study was approved by the Ethical Committee of University Hospital Brno, and all patients or their legal guardians provided informed consent for genetic testing.

### 2.2 Sequencing Analysis of Gene Deletion Breakpoints

To characterize gene deletions identified through NGS and CNV analysis, forward and reverse primer pairs were designed to amplify the predicted breakpoint regions of *NEB* and *RYR1*. Long‐range PCRs were performed using the UltraRun LongRange PCR Kit (Qiagen), and PCR products were sequenced via Sanger sequencing. Primer sequences are provided in the Supplementary Table S1.

## 3 Results

We conducted a retrospective analysis of patients with a clinical and genetic diagnosis of CM. A total of 79 unrelated patients were identified, each carrying CM‐related gene variants explaining their respective phenotypes. In total, 113 mutant alleles comprising 97 variants with a supposed pathogenic effect were detected. According to the Human Gene Mutation Database (HGMD 2025.1), 54 variants had been reported only in the Czech CM population to date.

The mode of inheritance was AD in 35 probands (44.3%), AR in 34 probands (43.0%), and XL in 10 probands (12.7%). The vast majority of variants were small‐scale mutations, with the exception of five large deletions. Two large gene deletions were identified in *MTM1* (the deletions of exons 1–4 and exon 15) and *NEB* (the deletion of exons 19–78 and exons 121–124); one large gene deletion was detected in *RYR1* (the deletion of exons 85–88).

The *RYR1* gene was the most common underlying gene, representing 39.2% of the total number of patients (31 of 79), followed by *ACTA1* (12/15.2%), *MTM1* (10/12.7%), *NEB* (8/10.1%), *TTN* (4/5.1%), *SCN4A* (3/3.8%), *MYBPC1* (3/3.8%), *TPM2* (2/2.5%), *TPM3* (2/2.5%), *CFL2* (1/1.3%), *DNM2* (1/1.3%), *LMOD3* (1/1.3%), and *TNNT3* (1/1.3%) (Table 1, Supplementary Figure S1). Supplementary Table S2 provides genotype–phenotype correlations, inheritance modes, and information on whether each variant has been previously reported in other CM patient populations. Supplementary Table S3 presents the classification of variants identified exclusively in Czech patients.

**TABLE 1:** Number of Probands in Individual CM‐Related Genes.

Gene	Inheritance	Number of probands	% of probands
*RYR1*	AR	17	21.5
*RYR1*	AD	14	17.7
*ACTA1*	AD	12	15.2
*MTM1*	XL	10	12.7
*NEB*	AR	7	8.9
*NEB*	AD	1	1.3
*TTN*	AR	4	5.1
*SCN4A*	AR	3	3.8
*MYBPC1*	AD	2	2.5
*MYBPC1*	AR	1	1.3
*TPM2*	AD	2	2.5
*TPM3*	AD	2	2.5
*CFL2*	AR	1	1.3
*DNM2*	AD	1	1.3
*LMOD3*	AR	1	1.3
*TNNT3*	AD	1	1.3

*RYR1*‐pathogenic variants were associated with AR inheritance in 17 probands and AD inheritance in 14 probands. A deletion encompassing exons 85–88 was identified in a patient with a known pathogenic variant in *trans* position. The clinical findings of AD *RYR1*‐related CMs were highly heterogeneous, ranging from mild nonprogressive myopathy occurring in multiple generations of studied families to severe muscle hypotonia with arthrogryposis multiplex congenita and skeletal deformities. The AD form usually begins in infancy or early childhood with muscle weakness, delayed motor development, and slow progression. Patients often walk independently but may struggle with running or climbing stairs. Common features include facial weakness, joint laxity, and skeletal issues like hip dislocation and scoliosis. Respiratory and cardiac problems are rare. In contrast, the AR form is characterized by severe hypotonia noticeable shortly after birth, generalized muscle weakness and atrophy, delayed motor development, and difficulties with walking. Patients often exhibit external ophthalmoplegia and bulbar muscle weakness, which can lead to feeding difficulties and respiratory insufficiency. Orthopedic complications such as joint laxity, contractures, hip dislocation, and scoliosis are frequent. Some individuals show prenatal symptoms including reduced fetal movements, polyhydramnios, and intrauterine growth restriction. AR cases tend to be more severe than AD.

Among the 12 *ACTA1*‐positive probands, one patient (Patient 11) carried a *de novo ACTA1* variant (c.1055C>T p.Ser352Phe), along with two additional pathogenic variants in *CRPPA* in *trans* position. According to OMIM, pathogenic variants in *CRPPA* are associated with muscular congenital dystrophy‐dystroglycanopathy with brain and eye anomalies and limb‐girdle muscular dystrophy‐dystroglycanopathy. The patient's phenotype included congenital hypotonia and muscle weakness; at 5 years of age, the inability to sit and walk was described together with joint hypermobility, pes equinovarus, and convergent strabismus; at 18 years of age, scoliosis, chest deformity, respiratory difficulties, malnutrition, and dilated cardiomyopathy were prominent; at this age there was a significant deterioration of the general condition and death due to cardiac and respiratory failure. Muscle biopsy at age 9 years revealed fibrous and adipose tissue replacement, but no significant structural changes or atrophic muscle fibers.

Among the 10 male probands with *MTM1*‐related XL myopathy, nine inherited pathogenic variants from asymptomatic mothers, while in one patient, maternal DNA was unavailable for analysis.

According to OMIM, pathogenic variants in *NEB* cause nemaline myopathy and arthrogryposis multiplex congenita; both diseases are associated with AR inheritance. We have seven patients with biallelic *NEB* variants with a supposed pathogenic effect, and one patient carrying the heterozygous deletion of exons 19–78 but without another detected variant in this gene. This deletion was identified in four members of the patient's family, all suffering from myopathy—the index patient, her two daughters, and the grandson (Supplementary Figure S2). This deletion is not predicted to change the translational reading frame. The first symptoms of our proband seen in the first decade included tripping, problems with running and jumping, fatigue, foot drop, generalized hypotonia, and areflexia. Slow progression followed. In the third decade, pelvifemoral muscle weakness developed, gradually followed by distal muscle weakness of the upper limbs. Loss of independent walking occurred at the age of 64 years, with tibioperoneal muscle weakness and distal muscle atrophies being the main symptoms. Furthermore, mild Achilles tendon contractures and mild restrictive respiratory disorder were present. The patient had no swallowing difficulties or scoliosis. Her two daughters had similar clinical manifestations—the first symptoms in the first decade (tripping, walking difficulties, recurrent ankle dislocations); at nearly 40 years of age, they had tibioperoneal muscle weakness and distal weakness and atrophies in the upper limbs, high arched feet, Achilles tendon contractures, mild restrictive respiratory disorders, and impaired fine motor skills. The grandson started walking at 1 year of age, but his gait was slightly atypical. At the age of 10, the tripping began, and weakness of the distal muscles was also present.

Additional patients with genetically confirmed CM and AR inheritance carried pathogenic variants in *TTN* (four probands), *SCN4A* (three probands), *MYBPC1*, *LMOD3*, and *CFL2* (one proband each). The AD form of CM was identified in patients with pathogenic variants in *TPM2, TPM3*, and *MYBPC1* (two patients each), *DNM2* and *TNNT3* (one patient each).

**FIGURE 1:**
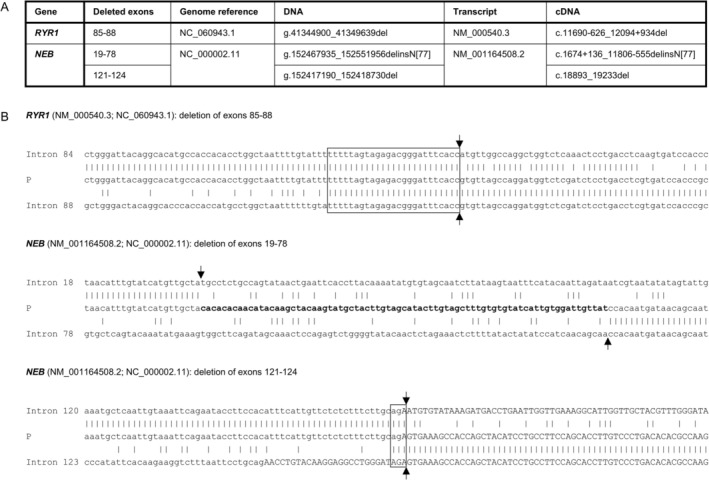
(A) Description of the multiexon deletions identified in the *RYR1* and *NEB* genes on the gDNA a cDNA level. (B) Visual representation of the breakpoint junctions in the *RYR1* and *NEB* genes. Exon sequences are in upper case letters, intron sequences are in lower case letters. Breakpoint sites are marked with vertical arrows. Regions of homology at the site of the junction are enclosed in boxes. Inserted nucleotides are in bold.

In order to reveal presumed mechanisms of structural variation genesis, we performed sequencing of individual breakpoint junctions in the *NEB* and *RYR1* deletions. Subsequently, we analyzed the extent of homology and the presence of repetitive elements in and around the breakpoints. Visual representation of the breakpoint regions is shown in Figure 1. The *RYR1* deletion encompassing exons 85–88 is 4.7 kb in size (c.11690‐626_12094+934del) and shows extensive sequence homology at the breakpoint junction. Two different deletions have been identified in the *NEB* gene. The first one, involving parts of exons 121 and 124, and exons 122 and 123, is 1.5 kb in size (c.18893_19233del), and the breakpoint junction shows microhomology of 3 bp. The second deletion involves exons 19–78, is 84 kb in size, and has 77 bp inserted at the deletion junction (c.1674+136_11806‐555delinsN[77]).

## 4 Discussion

This study provides a comprehensive genetic and clinical characterization of Czech patients with CM diagnosed between 2012 and July 2024, offering an updated perspective on the molecular landscape of the disease. The *RYR1* gene was the most common underlying gene, representing 39.2% of the total number of patients (31 of 79), followed by *ACTA1* (12 patients, 15.2%), *MTM1* (10 patients, 12.7%), *NEB* (eight patients, 10.1%), *TTN* (four patients, 5.1%), and others. We compared our results with three representative studies involving patients with pathogenic variants in CM‐related genes (Table 2). Natera‐de Benito et al. published the retrospective cross‐sectional data collection study of a clinical series with a diagnosis of CM, which was conducted at Hospital Sant Joan de Déu in Barcelona, Spain [9]. Data were collected from all CM patients followed up at the Neuromuscular Unit between 1990 and 2019. This cohort comprised 55 patients with genetically confirmed CM. *RYR1* was the most common underlying gene, representing 24 patients (43.6%), followed by *TTN* (eight patients, 14.5%), *MTM1* (seven patients, 12.7%), *NEB* (four patients, 7.3%), and others. Morales et al. analyzed DNA samples of patients followed at the French South‐West Reference Centre for Neuromuscular Disorders [10]. CM pathogenic variants were identified in 36 patients; the most frequent implicated gene was *RYR1* (10 patients, 27.8%), followed by *NEB* (seven patients, 19.4%), *TTN* (five patients, 13.9%), and *ACTA1* (five patients, 13.9%). Zhang et al. published the study encompassing patients with CM who were registered from 2007 to 2019 in the Department of Paediatrics, Peking University First Hospital, China [11]. Genetic diagnosis was confirmed in 26 of them; most often pathogenic variants occurred in *RYR1* (12 patients, 46.2%), followed by *ACTA1* (four patients, 15.4%), *NEB* (four patients, 15.4%), and *TTN* (three patients, 11.5%). The results of our study regarding the percentage representation of associated genes in the CM patient population are comparable to the above studies, which means that *RYR1* is most often associated with CM, followed by *ACTA1*, *MTM1*, *NEB*, and *TTN* genes, whose percentage representation may vary in different populations but is higher than other CM genes (Table 2).

**TABLE 2:** CM‐Related Genes in Selected Studies.

Population, the study	Number of patients with genetically confirmed CM	% of patients with confirmed genetics diagnosis				
		*RYR1*	*ACTA1*	*MTM1*	*NEB*	*TTN*
Czech, this study	79	39.2	15.2	12.7	10.1	5.1
Spanish, [9]	55	43.6	3.6	12.7	7.3	14.5
French, [10]	36	27.8	13.9	8.3	19.4	13.9
Chinese, [11]	26	46.2	15.4	—	15.4	11.5

Variants in the *RYR1* gene have been recognized as the most common cause of CMs. Both dominant and recessive variants have been reported in *RYR1*. Clinical heterogeneity was noted between patients with dominant and recessive *RYR1*‐related CMs, and within patients with the same inheritance mode. In the *RYR1* gene, we identified one large deletion associated with AR inheritance, the deletion of exons 85–88. So far, eight types of *RYR1* deletions involving one or more exons have been reported in HGMD; our deletion is new, not yet described.

Kiiski et al. published the first family with a dominantly inherited mutation of the *NEB* gene [12]. This ∼100 kb in‐frame deletion encompasses *NEB* exons 14–89, causing distal nemaline/cap myopathy in a three‐generation family. The mutated allele was shown to be expressed at the mRNA level and furthermore a deletion was shown to cause the production of a smaller mutant nebulin protein. Thus, it was suggested that this novel mutant nebulin protein has a dominant‐negative effect, explaining the first documented dominant inheritance of nebulin‐caused myopathy. The index patient, a young man, was more severely affected than his mother and grandmother. His first symptom was foot drop at the age of three. The course of the disease has been slowly progressive. On examination at the age of 30 years, he reported slight difficulties rising from the squatting position, grip strength having become weaker, and carrying heavy loads more difficult. He was able to climb stairs, albeit a bit slowly. He had moderate atrophy of the distal muscles of the upper and lower limbs, slight hypomimia, and high‐arched palate. Thus, our family carrying the heterozygous deletion of exons 19–78 is another described family carrying a dominant mutation in the *NEB* gene. We assume that, similar to the study by Kiiski et al. [12], our deletion is of in‐frame type, that is, not changing translation reading frame and causing the formation of a smaller mutant nebulin protein.

Estimated genesis of identified deletions in the *RYR1* and *NEB* genes was analyzed on the basis of sequence content of the particular breakpoints and surrounding areas. In the *RYR1* deletion c.11690‐626_12094+934del (exons 85–88), the breakpoint junction shows extensive sequence homology. The deletion probably originated from nonallelic homologous recombination (NAHR) between two *Alu* repeats: *AluSx* (g.41344686‐41344999) in intron 84 and *AluY* (g.41349442‐41349737) in intron 88 (coordinates in brackets are for T2T‐CHM13v2.0). *Alu* elements represent one of the most successful mobile elements, having a copy number well in excess of 1 million copies in the human genome (contributing almost 11% of the human genome) [13]. *Alu* repeat dispersion throughout the genome offers many opportunities for homologous recombinations and NAHR is the most common mechanism underlying disease associated genome rearrangements [14].

In the *NEB* deletions, c.1674+136_11806‐555delinsN[77] (exons 19–78) and c.18893_19233del (exons 121–124), the breakpoint junctions are characterized by no microhomology and 3 bp microhomology, respectively. We suppose that nonhomologous end joining (NHEJ), a homology‐independent pathway repairing DNA double‐strand breaks, is a mechanism of the deletion origin [15], but the presence of other alternative mechanisms producing genomic rearrangements, such as replication‐based mechanisms, cannot be excluded [16]. NHEJ generates breakpoint junctions, which can be characterized by simple blunt ends or microhomologies. In addition, small insertions have been identified in a number of junctions [15]. The deletion c.1674+136_11806‐555delinsN[77] is associated with the 77 bp insertion; this insertion showed no homology in the human genome. This deletion is dominantly inherited, suggesting it remains in‐frame. In contrast, the second *NEB* deletion of exons 121–124 is associated with recessive inheritance and likely disrupts the translational reading frame.

Advanced genomic technologies such as WGS, transcriptome (RNA) sequencing, and long‐read sequencing offer significant potential to improve the understanding of CM. WGS allows comprehensive detection of both small variants and large structural changes, including noncoding regions. RNA sequencing can reveal the functional impact of variants through analysis of gene expression and splicing. Long‐read sequencing provides better resolution of complex or repetitive regions and large deletions or insertions. Together, these methods can enhance diagnostic accuracy, clarify the pathogenicity of variants of uncertain significance, uncover novel pathogenic mechanisms, and improve genotype–phenotype correlations in CM.

In summary, we present 79 unrelated CM patients with genetically confirmed CM. Totally 113 mutant alleles carrying 97 different variants with a supposed pathogenic effect were identified. According to the HGMD database, 54 variants have been described only in the Czech CM population to date. All but five variants were small scale. Large gene deletions were identified in the *MTM1*, *NEB*, and *RYR1* genes. Sequencing of breakpoint junctions in the identified *NEB* and *RYR1* deletions allowed us to elucidate the upstream mechanisms leading to genomic instability and resulting in structural variations. Further, we present the family with dominant inheritance of the *NEB* deletion of exons 19–78. We assume that our family represents another reported case of a dominant mutation in the *NEB* gene and thus confirm that in some rare cases a large aberration in *NEB* may be dominant.

## Author Contributions

J.Z.: interpretation of identified genetic variants, study design, manuscript writing. B.L., L.M.: clinical monitoring of patients, evaluation of clinical and genetic findings. T.K., J.K., M.S.V., M.H.: molecular genetic diagnostics and interpretation of results. K.R.: bioinformatic processing of NGS data. J.H., M.R., R.M., P.D., L.J., T.H., M.M., M.K.: clinical monitoring of patients. J.Š., R.G., E.V., M.B., D.G., A.G., P.P., M.L., P.S., M.Š.: indications for genetic testing, genetic counseling. L.F.: study design; manuscript writing. All authors had full access to the data in the study; critically revised and approved the final version of the manuscript.

## Ethics Statement

This study involves human participants and was approved by the Ethical Committee of the University Hospital Brno (ID 4‐326/23/5). Participants gave informed consent to participate in the study before taking part.

## Conflicts of Interest

The authors declare no conflicts of interest.

## Peer Review

The peer review history for this article is available at https://www.webofscience.com/api/gateway/wos/peer‐review/10.1111/cge.14782.

## Supporting information


**Figure S1.** Graphical representation of number of the probands in individual CM‐related genes and by inheritance type.
**Figure S2.** A pedigree of the family with a dominant *NEB* deletion of exons 19–78.

**Table S1.** Primers used for breakpoint analysis/confirmation of large deletions.

**Table S2.** Genotype and phenotype of Czech patients with congenital myopathy.

**Table S3.** Classification of variants identified only in Czech patients with congenital myopathy.

## Data Availability

The data on the basis of which the publication was created are available at the Centre of Molecular Biology and Genetics, University Hospital Brno, Jihlavská 20, Brno 625 00, Czech Republic.
