# Supplementary material for: Genetic and Structural Variations in Czech Patients With Congenital Myopathies
Source: Clin Genet. 2025 Jun 17;108(6):678–83. doi: 10.1111/cge.14782 (PMC12580487; doi:10.1111/cge.14782)

Figure S1. Graphical representation of number of the probands in individual CM-related genes (A) and by inheritance type (B)

A)

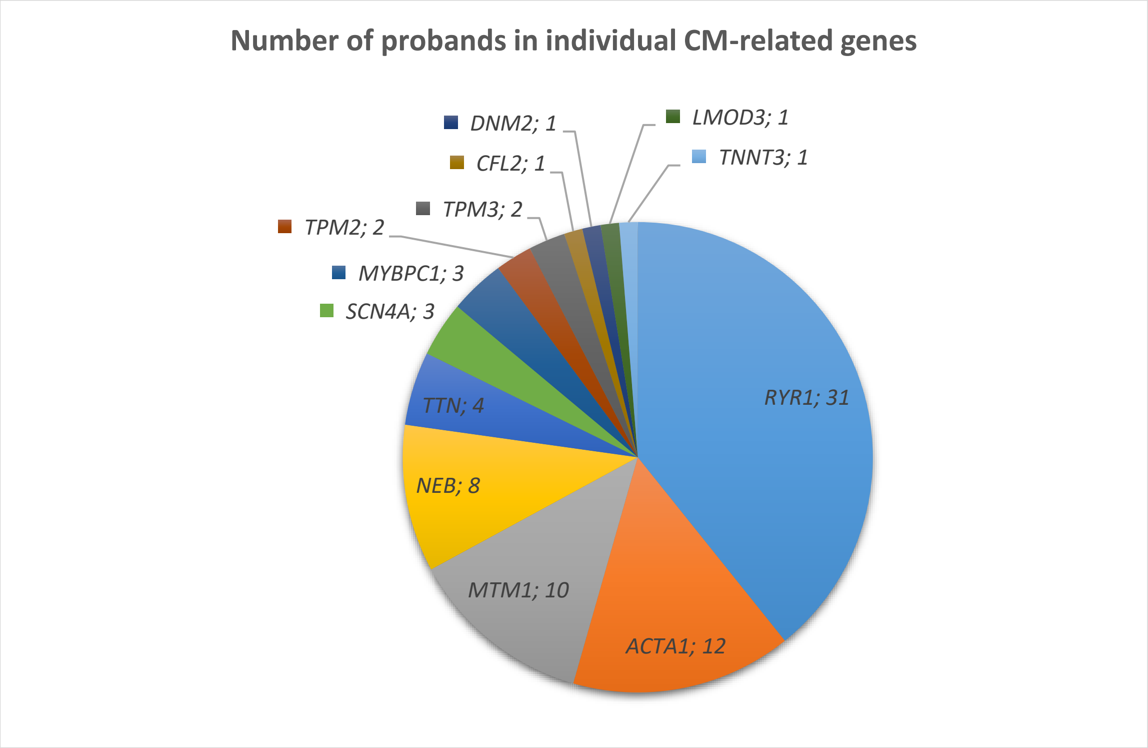

B)

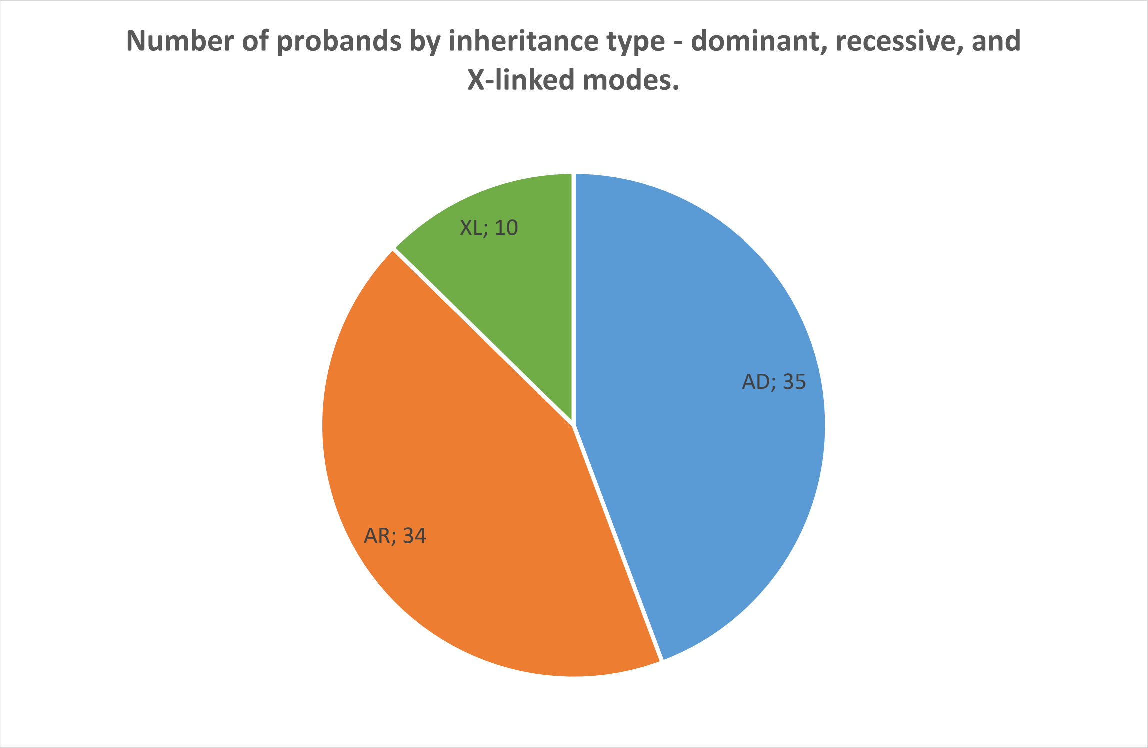

AD – autosomal dominant, AR - autosomal recessive, XL - X-linked.

Figure S2. A pedigree of the family with a dominant *NEB* deletion of exons 19-78

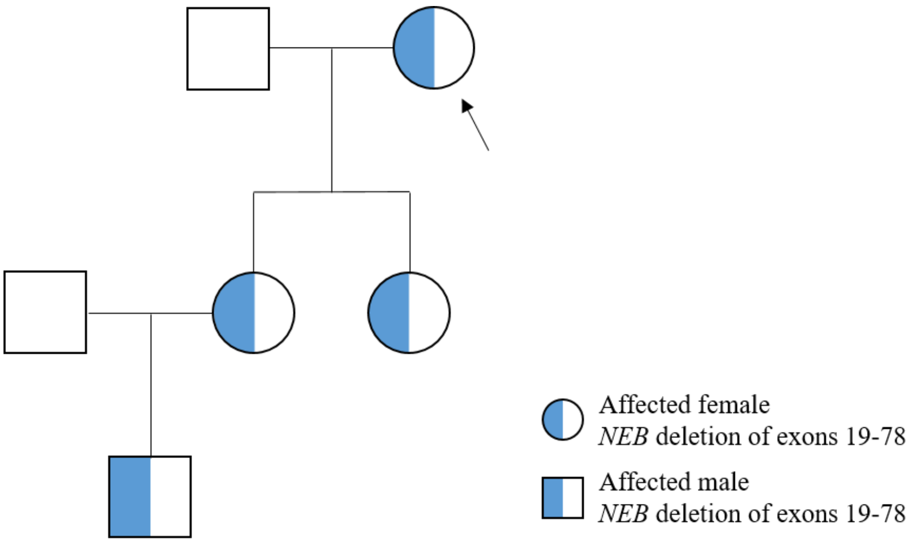

Supplement: Supplementary file 1 — Figure S1. Graphical representation of number of the probands in individual CM‐related genes and by inheritance type. Figure S2. A pedigree of the family with a dominant NEB deletion of exons 19–78. [file CGE-108-678-s004.pdf]
