# Supplementary material for: Genetic and Structural Variations in Czech Patients With Congenital Myopathies
Source: Clin Genet. 2025 Jun 17;108(6):678–83. doi: 10.1111/cge.14782 (PMC12580487; doi:10.1111/cge.14782)
Supplement: Supplementary file 2 — Table S1. Primers used for breakpoint analysis/confirmation of large deletions. [file CGE-108-678-s001.docx]

**Table S1. Primers used for breakpoint analysis / confirmation of large deletions**

|  | **Primer sequence** | **Primer genomic coordinates** | **Ta** |
| --- | --- | --- | --- |
| ***RYR1* - exon 85-88del** |  | NC_000019.9 | 60 |
| F primer | gagagaaggtcatggcggat | 39028519-39028539 |  |
| R primer | gattccacgagcatgtccac | 39038907-39038927 |  |
| Sequencing primer | cccagagcctagaatactgca | 39032768-39032789 |  |
| ***NEB* - exon 19-78del** |  | NC_000002.11 | 58 |
| F primer | ggcctacccaagctctagat | 152552307-152552327 |  |
| R primer | gtgtttgaggaaggtaataggcc | 152467171-152467194 |  |
| ***NEB* - exon 121-124del** |  | NC_000002.11 | 60 |
| F primer | cagctggtgagaggcctac | 152418838-152418857 |  |
| R primer | ttcttggagtggagagcagg | 152417038-152417058 |  |

Ta – annealing temperature / °C
